# Supplementary material for: Neoadjuvant Chemoradiotherapy versus Chemotherapy for Gastroesophageal Junction Adenocarcinoma; Which Is the Optimal Treatment Option?
Source: Cancers (Basel). 2022 Nov 28;14(23):5856. doi: 10.3390/cancers14235856 (PMC9736946; doi:10.3390/cancers14235856)
Supplement: Supplementary file 1 [file cancers-14-05856-s001.zip › cancers-2001048-supplementary.pdf]

# Supplementary Materials: Neoadjuvant Chemoradiotherapy Versus Chemotherapy for Gastroesophageal Junction Adenocarcinoma; Which Is the Optimal Treatment Option?

**Eric Zandirad, Hugo Teixeira Farinha, Beatriz Barberá–Carbonell, Sandrine Geinoz, Nicolas Demartines, Markus Schäfer and Styliani Mantziari**

**Table S1.** Logistic regression analysis for complete pathologic response (TRG1).

| | **Unadjusted OR** | **95%CI** | ***P*–value** |
|---|---|---|---|
| % Baseline weight loss | 1.03 | 0.88–1.19 | 0.669 |
| cT stage | | | |
| 2 | 1 | | |
| 3–4 | 0.34 | 0.06–2.62 | 0.236 |
| cN stage | | | |
| 0 | 1 | | |
| 1 | 0.34 | 0.07–1.59 | 0.158 |
| 2–3 | 1.50 | 0.24–8.52 | 0.644 |
| Differenciation grade | | | |
| G1 | 1 | | |
| G2 | 0.75 | 0.09–16.0 | 0.811 |
| G3 | 0.22 | 0.02–5.39 | 0.259 |
| Baseline SUVmax | 0.98 | 0.88–1.07 | 0.796 |
| Signet–ring histology | 0.41 | 0.02–2.44 | 0.419 |
| HER2+ status | 1.50 | 0.16–14.2 | 0.707 |
| NAT type | | | |
| RCT | 1 | | |
| CT | 0.518 | 0.07–2.20 | 0.423 |

As only cN status had $p < 0.2$ in univariate analysis (simple logistic regression), multivariate analysis was not performed.

**Table S2.** Cox regression analysis for Overall Survival (OS).

| | **Unadjusted OR** | **95%CI** | ***P*–value** | **Adjusted OR** | **95%CI** | ***P*–value** |
|---|---|---|---|---|---|---|
| % Baseline weight loss | 1.02 | 0.93–1.11 | 0.698 | | | |
| cT stage | | | | | | |
| 2 | 1 | | 0.652 | | | |
| 3 | 1.59 | 0.21–12.2 | | | | |
| cN stage | | | | | | |
| 0 | 1 | | | | | |
| 1 | 1.48 | 0.42–5.21 | 0.538 | | | |
| 2–3 | 0.54 | 0.05–5.23 | 0.597 | | | |
| SUVmax | 0.96 | 0.89–1.03 | 0.301 | | | |
| Signet–ring histology | 0.76 | 0.22–2.59 | 0.665 | | | |
| HER2+ status | 0.24 | 0.02–2.45 | 0.23 | | | |
| NAT type | | | | | | |
| RCT | 1 | | 0.269 | | | |
| CT | 0.50 | 0.14–1.71 | | | | |
| RCT dose | | | | | | |
| 41.4Gy | 1 | | | | | |
| 45Gy | 2.24 | 0.57–8.79 | 0.246 | | | |
| 50.4Gy | 2.22 | 0.57–8.62 | 0.248 | | | |
| pT status | | | | | | |
| 0 | 1 | | | 1 | | |

| | | | | | | |
|---|---|---|---|---|---|---|
| 1 | 3.08 | 0.31–30.3 | 0.334 | 1.08 | 0.29–29.4 | 0.358 |
| 2 | 5.89 | 0.66–52.9 | 0.113 | 1.36 | 0.41–31.17 | 0.232 |
| 3 | 3.13 | 0.40–24.3 | 0.276 | 0.73 | 0.25–17.01 | 0.494 |
| 4 | 6.15 | 0.37–102.2 | 0.205 | 1.86 | 0.32–126.8 | 0.221 |
| cN status | | | | | | |
| 0 | 1 | | | 1 | | |
| 1 | 2.10 | 0.68–6.52 | 0.197 | 2.21 | 0.68–7.19 | 0.189 |
| 2 | 2.71 | 0.92–7.94 | 0.069 | 4.00 | 1.22–13.17 | 0.022 |
| 3 | 0.97 | 0.12–7.92 | 0.981 | 1.32 | 0.13–13.10 | 0.811 |
| pCR | 0.274 | 0.03–2.07 | 0.210 | | | |
| R1 resection | 1.60 | 0.51–5.04 | 0.422 | | | |
| Major Complications | 2.57 | 1.09–6.06 | 0.031 | 1.15 | 1.21–8.12 | 0.018 |

Variables with $p < 0.2$ in univariate analysis were included in the multivariate model.

**Table S3.** Cox regression analysis of Disease–Free Survival (DFS).

| | **Unadjusted OR** | **95%CI** | **P–value** | **Adjusted OR** | **95%CI** | ***P*–value** |
|---|---|---|---|---|---|---|
| cT status | | | | | | |
| 2 | 1 | | 0.334 | | | |
| 3 | 2.01 | 0.49–8.35 | | | | |
| cN status | | | | | | |
| 0 | 1 | | | | | |
| 1 | 1.32 | 0.60–2.91 | 0.484 | | | |
| 2–3 | 1.46 | 0.53–4.04 | 0.465 | | | |
| Differenciation grade | | | | | | |
| 1 | 1 | | | 1 | | |
| 2 | 1.68 | 0.39–7.28 | 0.485 | 1.19 | 0.25–5.75 | 0.821 |
| 3 | 2.89 | 0.67–12.4 | 0.153 | 1.97 | 0.42–9.18 | 0.387 |
| SUVmax | 1.01 | 0.97–1.05 | 0.635 | | | |
| Signet–ring cell histology | 1.39 | 0.69–2.82 | 0.359 | | | |
| HER2+ status | 0.38 | 0.09–1.49 | 0.167 | | | |
| NAT type | | | | | | |
| RCT | 1 | 0.47–1.71 | 0.753 | | | |
| CT | 0.903 | | | | | |
| pT status | | | | | | |
| 0 | 1 | | | 1 | | |
| 1 | 0.25 | 0.05–1.27 | 0.094 | 0.19 | 0.03–1.12 | 0.066 |
| 2 | 0.95 | 0.27–3.38 | 0.937 | 0.52 | 0.09–2.95 | 0.466 |
| 3 | 1.04 | 0.44–2.50 | 0.919 | 0.46 | 0.13–1.58 | 0.219 |
| 4 | 6.32 | 1.49–26.8 | 0.012 | 0.91 | 0.06–13.9 | 0.947 |
| pN status | | | | | | |
| 0 | 1 | | | 1 | | |
| 1 | 2.49 | 1.16–5.36 | 0.019 | 1.94 | 0.74–5.10 | 0.177 |
| 2 | 3.55 | 1.67–7.55 | <0.001 | 3.32 | 1.32–8.35 | 0.011 |
| 3 | 4.61 | 1.94–10.9 | <0.001 | 4.85 | 1.25–18.8 | 0.022 |
| TRG1 | 1.09 | 0.46–2.57 | 0.848 | | | |
| R1 resection | 2.40 | 1.22–4.71 | 0.011 | 1.28 | 0.49–3.34 | 0.614 |
| Major complica-tions | 0.64 | 0.34–1.21 | 0.172 | 0.741 | 0.34–1.62 | 0.452 |

Variables with $p < 0.2$ in univariate analysis were included in the multivariate model.
